# Exploring Predictive Factors for Heart Failure Progression in Hypertensive Patients Based on Medical Diagnosis Data from the MIMIC-IV Database

**DOI:** 10.3390/bioengineering11060531

**Published:** 2024-05-23

**Authors:** Jinmyung Jung, Doyoon Kim, Inkyung Hwang

**Affiliations:** Division of Data Science, College of Information and Communication Technology, The University of Suwon, Hwaseong 18323, Republic of Korea; doyoon510@suwon.ac.kr (D.K.); dlsrud1017@suwon.ac.kr (I.H.)

**Keywords:** heart failure, hypertension, predictive factors, MIMIC-IV database, data mining, XGBoost modeling, chi-square test

## Abstract

Heart failure is associated with a significant mortality rate, and an elevated prevalence of this condition has been noted among hypertensive patients. The identification of predictive factors for heart failure progression in hypertensive individuals is crucial for early intervention and improved patient outcomes. In this study, we aimed to identify these predictive factors by utilizing medical diagnosis records for hypertension patients from the MIMIC-IV database. In particular, we employed only diagnostic history prior to hypertension to enable patients to anticipate the onset of heart failure at the moment of hypertension diagnosis. In the methodology, chi-square tests and XGBoost modeling were applied to examine age-specific predictive factors across four groups: AL (all ages), G1 (0 to 65 years), G2 (65 to 80 years), and G3 (over 80 years). As a result, the chi-square tests identified 34, 28, 20, and 10 predictive factors for the AL, G1, G2, and G3 groups, respectively. Meanwhile, the XGBoost modeling uncovered 19, 21, 27, and 33 predictive factors for these respective groups. Ultimately, our findings reveal 21 overall predictive factors, encompassing conditions such as atrial fibrillation, the use of anticoagulants, kidney failure, obstructive pulmonary disease, and anemia. These factors were assessed through a comprehensive review of the existing literature. We anticipate that the results will offer valuable insights for the risk assessment of heart failure in hypertensive patients.

## 1. Introduction

Hypertension is a prevalent condition, impacting three out of every ten adults [1]. Hypertension is dangerous because it leads to complications like arteriosclerosis, stroke, and heart failure. Heart failure (HF) is a disease in which the body is not supplied with the amount of blood required due to impaired diastolic or systolic function of the heart. On average, HF patients have a one-year mortality rate of 33%, indicating a very poor prognosis [2]. Crucially, the incidence of HF is about three times higher in hypertensive patients than in nonhypertensive populations [3]. Therefore, patients with hypertension need to be especially careful to avoid developing HF. We believe that identifying predictive factors for HF progression in hypertensive patients would be of great benefit.

The purpose of this study is to identify predictive factors for heart failure progression in hypertensive patients, and for this purpose, it was decided to use diagnosed medical conditions. Genetic data such as gene expression and DNA sequences can also be used to identify the predictive factors; however, genetic data on patients with hypertension and HF are not sufficient to build analytical models. Furthermore, even if analytical models are successfully constructed using genetic data, hypertension patients will need to obtain their genetic information in order to utilize the models. Predictive factors based on medical conditions, which are easily accessible to most hypertension patients, are expected to be highly useful.

Importantly, only medical histories recorded prior to the time of hypertension diagnosis were employed in this study. This will make it possible to predict heart failure at the exact point of hypertension diagnosis based on previous medical conditions. The reason for establishing this strategy is because preliminary analysis confirmed that most patients develop heart failure within a year after being diagnosed with hypertension, which represents a very rapid progression (refer to Section 2.2 for more details).

There are many well-known predictive factors for heart failure derived from medical conditions, which can be broadly divided into two categories, i.e., cardiac dysfunctions and adult diseases [4]. Coronary artery disease and valvular heart disease can be included in cardiac dysfunctions [5,6], and type 2 diabetes and obesity are associated with adult diseases [7,8]. Hypertension, known as a representative adult disease, is also one of the well-known predictive factors for HF [9]. However, to our knowledge, this study is the first to identify predictors of HF in the setting of diagnosed hypertension. We hope that the results of this study will be of great help to hypertensive patients.

**Figure 1:**
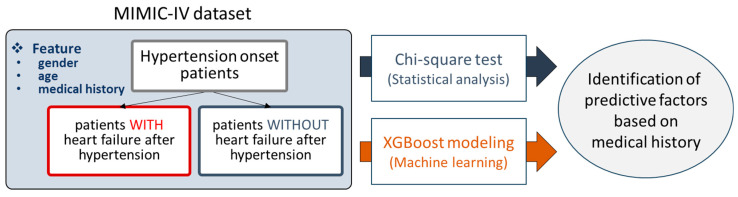
Research outline.

For this study, we decided to use the MIMIC-IV database, containing various kinds of clinical data such as diagnosed diseases and demographic information. Patients diagnosed with hypertension were selected from the MIMIC-IV dataset and divided into two groups: those who later developed HF and those who did not. For each patient, medical history prior to the point of hypertension diagnosis was processed and obtained, and age and gender information were added as well. Then, two analysis methods were applied to the preprocessed data, i.e., the chi-square test and XGBoost modeling. In the chi-square test, statistically significant medical conditions were characterized as predictive factors. By training XGBoost models, the feature importance scores of the trained models were employed to reveal predictive factors (Figure 1).

**Figure 2:**
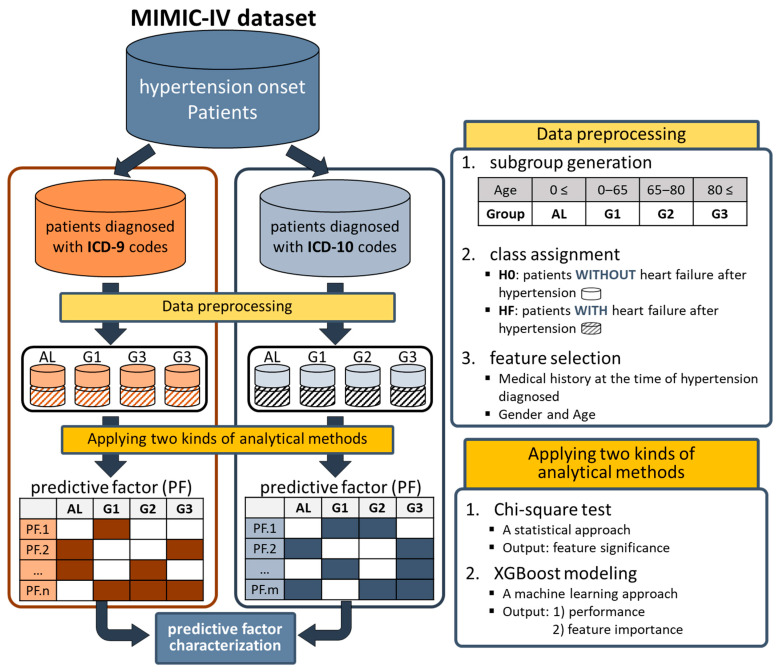
Strategy overview. Patients diagnosed with hypertension from the MIMIC-IV dataset were grouped based on ICD systems (9 and 10). Data preprocessing involved three steps: (1) subgroup generation, (2) class assignment, and (3) feature selection. It resulted in four preprocessed subgroups for each ICD system. The preprocessed data underwent analysis using a chi-square test and XGBoost modeling. Predictive factors were characterized as medical conditions proven to be significant by the chi-square test or scored as high feature importance by XGBoost modeling. Finally, the predictive factors consistently characterized across the both ICD systems were considered overall predictive factors for heart failure progression in hypertensive patients.

We also prepared a detailed strategy overview for this study, shown in Figure 2. The prepared hypertensive patients were divided into two groups according to the ICD systems used (9 and 10), because both ICD systems were used simultaneously in the MIMIC-IV database. Furthermore, they were further divided into four subgroups according to age: AL (all ages), G1 (0 to 65 years), G2 (65 to 80 years), and G3 (over 80 years). By applying both chi-square test and XGBoost modeling to the subgroups and integrating their outcomes, we characterized the 21 overall predictive factors, such as atrial fibrillation, the use of anticoagulants, kidney failure, pneumonia, and anemia. Then, they were assessed through an extensive review of the literature.

The manuscript begins with introduction outlining the importance of identifying predictive factors for heart failure progression in hypertensive patients. Following this, the materials and methods section details the utilization of the MIMIC-IV dataset, data preprocessing techniques, and analytical methods such as the chi-square test and XGBoost modeling. The results section presents findings from the chi-square test, XGBoost modeling, and overall predictive factor characterization. Subsequently, an evaluation of the results is conducted through a review of the literature, leading to a discussion and conclusions section. We assert that the following three aspects are the innovative contributions of this study: (1) Identification of predictive factors that anticipate the onset of heart failure at the time of hypertension diagnosis; (2) Integration of two analytical methods, the chi-square test and XGBoost modeling, to examine heart failure predictive factors; (3) Identification of heart failure predictive factors across four distinct age groups.

## 2. Materials and Methods

### 2.1. MIMIC-IV Dataset

In this study, we decided to use the MIMIC-IV (Medical Information Mart for Intensive Care IV) dataset, which is a comprehensive and widely utilized resource in the field of healthcare research [10,11]. The MIMIC-IV dataset contains deidentified electronic health records from patients admitted to the Beth Israel Deaconess Medical Center in Boston. It provides a rich and diverse collection of clinical data, including diagnosed diseases, laboratory results, medications, and demographic information, spanning over a decade [12,13]. Researchers can leverage this dataset to handle various medical issues, such as predicting patient outcomes and understanding disease trajectories [14,15]. Among all patients in MIMIC-IV, only data on patients diagnosed with hypertension (code 4019 for ICD-9 and I10 for ICD-10) were collected. As two types of ICD systems (9 and 10) were used for diagnosis in the MIMIC-IV database, we classified the patients into two groups based on the ICD systems used (top of Figure 2). During this process, patients using both ICD systems were excluded.

### 2.2. Data Preprocessing

The three steps of data preprocessing were performed sequentially on the prepared data (top right side of Figure 2): (1) subgroup generation; (2) class assignment; and (3) feature selection. Firstly, subgroups were generated based on age at the first diagnosis of hypertension (FDH), allowing four kinds of subgroups, i.e., AL (0 ≤ age, entire data), G1 (0 ≤ age < 65), G2 (65 ≤ age < 80), and G3 (80 ≤ age). Secondly, patients in each subgroup were divided into two classes (H0 and HF). Class H0 was assigned to patients without heart failure after the FDH, and class HF was assigned to patients diagnosed with heart failure after the FDH. The ICD codes in Table S1 were used to determine which patients were diagnosed with HF. During this process, patients diagnosed with HF before the FDH were excluded. The number of patients in H0 and HF classes for each subgroup is summarized in Table 1. Thirdly, features used in analytical methods were selected. Medical conditions diagnosed before FDH (i.e., the previous medical history at the time of FDH) were considered to be candidate features. We noticed that the number of medical conditions diagnosed before FDH was very large: more than 5000 in ICD-9 and more than 8000 in ICD-10. Therefore, for each group, only frequently diagnosed medical history data in patients of class HF (≥5%) were selected as the features for analytical methods. Additionally, two pieces of personal information, gender and age, were added.

**Figure 3:**
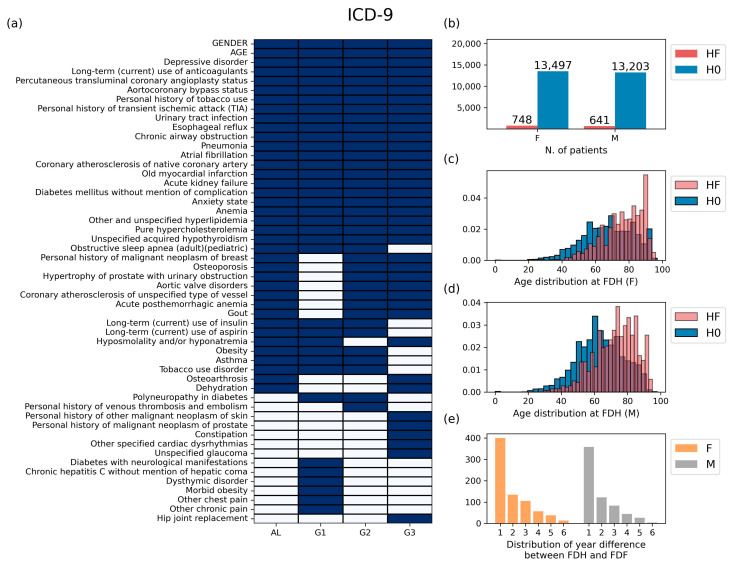
Basic statistics of the preprocessing data for patients using the ICD-9 system. (**a**) The selected features of each group for the analysis process. (**b**) The number of patients by gender. (**c**,**d**) The distribution of ages at the first diagnosis of hypertension (FDH) for (**c**) male patients and (**d**) female patients. (**e**) The distribution of year difference between FDH and FDF. HF: group of patients diagnosed with heart failure after hypertension, H0: group of patients without heart failure after hypertension, FDH: age at the first diagnosis of hypertension, FDF: age at the first diagnosis of heart failure.

The basic statistics for the data prepared through the preprocessing stage are shown in Figure 3 for ICD-9 and Figure S1 for ICD-10. In the ICD-9 case, the number of features selected in at least one group was 52, and the number of features selected in all four groups was 22 (Figure 3a). The selected ICD codes and their full names are displayed in Table S2. Patients belonging to class HF account for 5.25% in the female group and 4.63% in the male group (Figure 3b). In both genders, the FDH distribution of class HF was more skewed to the right compared to that of class H0, indicating that the older the age of FDH presentation, the more likely patients are to develop heart failure later in life (Figure 3c,d). We also found that the first diagnosis of heart failure (FDF) most frequently occurred within one year after FDH (Figure 3e). The same kind of information corresponding to ICD-10 is provided in Figure S1 and Table S3.

### 2.3. Analytical Methods

#### 2.3.1. Chi-Square Test

Two kinds of analysis methods were used in this study: the chi-square test and XGBoost modeling. Chi-squared is a statistical test used to assess the association between categorical variables. It compares the observed distribution of data with the expected distribution, assuming that there is no significant relationship between the variables. Chi-squared tests are commonly employed in various fields, including biology and medical science, to examine the dependence of categorical variables [16]. When using the chi2_contingency function in the Python scipy package (https://docs.scipy.org/doc/scipy/reference/generated/scipy.stats.chi2_contingency.html accessed on 22 May 2024), the chi-square test is applied between the class features, consisting of HF and H0, and each of the selected medical histories.

#### 2.3.2. XGBoost Modeling

Among various machine learning models including decision trees, support vector machines, random forest, and logistic regression, we decided to use eXtreme Gradient Boosting (XGBoost) to predict heart failure progression, which is intensively used and shows outstanding performance in the biomedical field [17,18,19,20]. XGBoost is an ensemble learning method that combines multiple decision trees to create a robust and accurate predictive model. It is a powerful and versatile machine learning algorithm that has gained widespread popularity for its exceptional performance across various predictive modeling tasks [21].

In this study, XGBoost models that classified the class feature consisting of HF and H0 were trained based on gender, age, and the medical history selected in each group, implemented using the Python xgboost package with default parameters (https://xgboost.readthedocs.io/en/stable/python/ accessed on 22 May 2024). For each of the four groups, the XGBoost models were generated 1000 times with randomly sampled balanced datasets. In more detail, to build a single XGBoost model, a merged dataset was prepared by concatenating patients in class HF and a portion of patients in class H0 that were randomly sampled as many as the patients in class HF. Then, 80% of the merged data were used for training, and the remaining data were used for testing.

In this study, two types of outputs were extracted from the trained XGBoost models, i.e., area under the ROC curve (AUC) and feature importance (FI). AUC is one of the most frequently used performance metrics in machine learning modeling, and it has a higher value when a model predicts test samples more accurately. FI was assigned to each feature during the model training process, which indicates the extent of performance reduction when a certain feature is perturbed. A feature exhibiting a high FI suggests its critical role in class discrimination [19]. To determine significant predictive factors by FI, we computed empirical *p*-values for FIs because there is not a conventional cutoff for determining significance. To this end, we constructed a background distribution of FIs and decided to use 0.0251 as the significance cutoff, with an empirical *p*-value of 0.01.

## 3. Results

### 3.1. Chi-Squared Test

**Figure 4:**
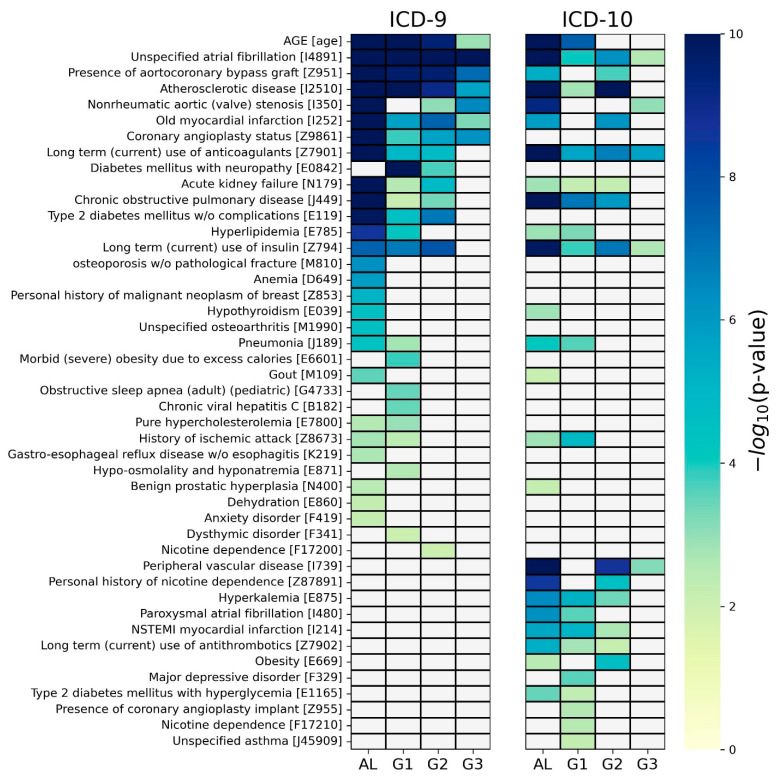
The predictive factors identified by chi-squared tests. For each of the two ICD systems, the *p*-values of the predictive factors identified by the chi-squared tests are visualized as a heatmap with the negative logarithm format (base 10). Only predictive factors showing significance (*p*-value < 0.01) are displayed in the colors on the color bar, while nonsignificant factors are grayed out. For simplicity of presentation, factors identified in the ICD-9 system were converted to ICD-10 codes. Detailed results are shown in Table S4 for ICD-9 and Table S5 for ICD-10.

For each of the four groups, a chi-square test was applied between the class feature and each piece of medical history information considered in the corresponding group. The significant predictive factors (*p*-value < 0.01) are depicted in a heatmap for each ICD system in Figure 4, where nonsignificant factors are grayed out. As shown on the left side of Figure 4, for the case of ICD-9, 33 predictive factors were identified as significant in at least one group, and six predictive factors were identified in all four groups (i.e., age, atrial fibrillation, aortocoronary bypass graft, atherosclerosis disease, old myocardial infarction, and coronary angioplasty status). A total of 26, 21, 14, and 7 predictive factors were determined in AL, G1, G2, and G3 groups, respectively. We noticed that there were several group-specific predictive factors in each group, e.g., anemia for AL, obstructive sleep apnea for G1, and nicotine dependence for G2. Detailed results are shown in Table S4.

As shown on the right side of Figure 4, for the case of ICD-10, 28 predictive factors were identified as significant in at least one group. In addition, three predictive factors were identified in all four groups (i.e., unspecified atrial fibrillation, long-term use of anticoagulants, and long-term use of insulin). Overall, 24, 19, 14, and 5 predictive factors were determined in AL, G1, G2, and G3 groups, respectively. We also noticed that there were several group-specific predictive factors in each group, e.g., hypothyroidism for AL and major depressive disorder for G1. Detailed results are presented in Table S5.

### 3.2. XGBoost Modeling

**Figure 5:**
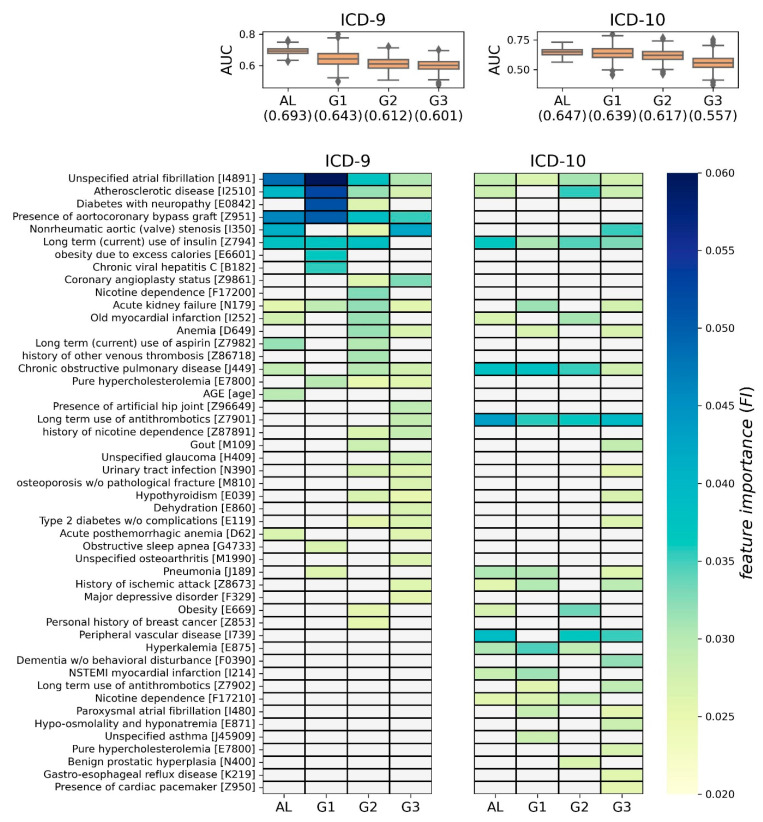
Predictive factors by XGBoost modeling. For each subgroup, the AUCs of the 1000 trained XGBoost models are shown as a boxplot along with their averages. Furthermore, the feature importance (FI) obtained from the trained XGBoost models is visualized as a heatmap, where a value in each cell represents the averaged FI (Ave.FI) of the 1000 XGBoost models. In the heatmap, only significant factors (Ave.FI > 0.0251) are displayed in the color on the color bar, while nonsignificant factors are grayed out. A significance cutoff of 0.0251 was determined with an empirical *p*-value of 0.01 based on a background distribution of FIs. For simplicity of presentation, factors identified in the ICD-9 system were converted to ICD-10 codes. Detailed results are depicted in Table S6 for ICD-9 and Table S7 for ICD-10.

For each of the four groups, 1000 XGBoost models were generated. Then, their AUCs and FIs were depicted as boxplots and heatmaps, respectively (Figure 5). In the Supplementary Material, the average precision rate (APR) of the XGBoost models are also shown as boxplots in Figure S2. Each cell in the heatmap represents the averaged FI (Ave.FI) of the 1000 XGBoost models, and Ave.FI is colored based on the color bar if significant (>0.0251); otherwise, it is grayed out (refer to Section 2.3.2 for more details).

In the case of ICD-9, the average of 1000 AUC was highest in the AL group at 0.693, and lowest in the G3 group at 0.601. From a volatility perspective, the models in the AL group were the most stable, and those in the G1 group were associated with the largest variation. Using FI, 36 predictive factors were identified as significant in at least one group, and 4 predictive factors were identified in all four groups (i.e., atrial fibrillation, atherosclerotic heart disease, aortocoronary bypass graft, and acute kidney failure). Overall, 11, 11, 22, and 22 predictive factors were determined in AL, G1, G2, and G3 groups, respectively. We noticed that there were several group-specific predictive factors in each group, e.g., age for AL, obesity for G1, gout for G2, and long-term use of anticoagulants for G3 (left side of Figure 5).

In the case of ICD-10, the average of 1000 AUC was highest in the AL group, at 0.647, and lowest in the G3 group at 0.557. From a volatility perspective, the models in the AL group were the most stable, and those in the G1 and G3 groups were associated with the largest variation. Using FI, 29 predictive factors were identified as significant in at least one group, and 4 predictive factors were identified in all four groups (i.e., long-term use of anticoagulants, chronic obstructive pulmonary disease, long-term use of insulin, and unspecified atrial fibrillation). Overall, 13, 14, 11, and 22 predictive factors were determined in AL, G1, G2, and G3 groups, respectively. We noticed that there were several group-specific predictive factors in each group, e.g., asthma for G1, benign prostatic hyperplasia for G2, and dementia without behavioral disturbance for G3 (right side of Figure 5).

### 3.3. Overall Predictive Factor Characterization

For each analytical method, predictive factors consistently characterized across both ICD systems were determined as overall predictive factors. This approach will help to reduce false positives and increase accuracy. Hence, each of the four subgroups possessed two sets of the overall predictive factors derived from chi-squared test and XGBoost modeling. To this end, the ICD-9 codes were converted to ICD-10 codes with the help of an online conversion program (https://www.icd10data.com/Convert accessed on 22 May 2024).

**Figure 6:**
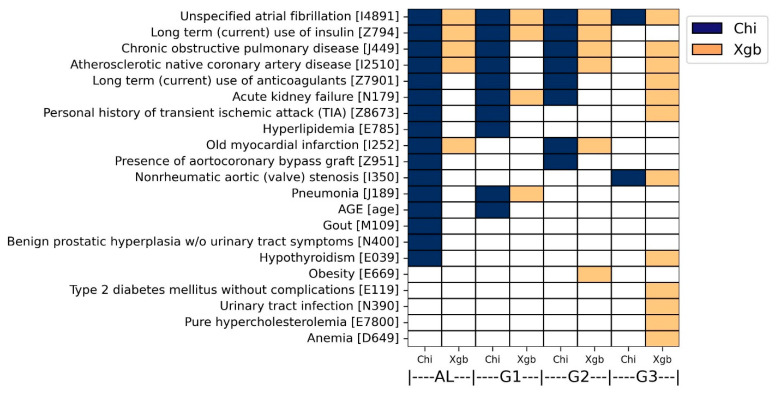
Overall predictive factors of heart failure progression in hypertensive patients for each of the two analytical methods. For each analytical method, overall predictive factors were determined as the predictive factors consistently characterized across both ICD systems. Therefore, each of the four subgroups has two lists of the overall predictive factors determined through the chi-squared test and XGBoost modeling.

As a result, 21 overall predictive factors were finally characterized by both analyses. More specifically, in the chi-square analysis, 16, 10, 8, and 2 predictive factors were commonly identified in AL, G1, G2, and G3 groups, respectively (colored dark blue in Figure 6). Atrial fibrillation (ICD-10: I4891) was determined by both ICD systems in all four groups. In addition, five diseases were determined by both ICD systems in three groups, including acute kidney failure (ICD-10: N179), atherosclerotic native coronary artery disease (ICD-10: I2510), long-term use of anticoagulants (ICD-10: Z7901), long-term use of insulin (ICD-10: Z794), and chronic obstructive pulmonary disease (ICD-10: J449).

In the XGBoost analysis, 5, 4, 6, and 12 predictive factors were commonly identified in AL, G1, G2, and G3 groups, respectively (colored orange in Figure 6). Atrial fibrillation (ICD-10: I4891) was determined by both ICD systems in all four groups. In addition, three diseases were determined by both ICD systems in three groups: chronic obstructive pulmonary disease (ICD-10: J449), atherosclerotic native coronary artery disease (ICD-10: I2510), and long-term use of insulin (ICD-10: Z794). The overall predictive factors are also described in Table S8.

## 4. Evaluation

The overall predictive factors were assessed by exploring the evidence in the literature. Firstly, atrial fibrillation, selected from all four groups, is known to be one of the diseases that promotes the formation of blood clots in the atria [22], which is a well-known factor that contributes to HF by impairing blood flow [23]. The use of anticoagulants is part of the treatment to reduce blood clots, rather than a cause of HF. Transient ischemic attack can also occur due to blood clot formation, similar to HF, rather than being a predictive factor for heart failure [24].

Similar to blood clots, elevated levels of lipids or glucose in the bloodstream can cause arteries to narrow and harden, impairing blood flow to the heart muscles [25]. Among the overall predictive factors associated with this phenomenon are hyperlipidemia, hypercholesterolemia, diabetes, and the use of insulin.

Several medical conditions related to weakened heart function have been identified as predictive factors, including coronary artery disease (CAD), coronary bypass graft surgery, and aortic valve stenosis. Coronary arteries are blood vessels that supply oxygen and nutrients to the heart muscles, and CAD is a disease that causes a narrowing of the coronary arteries. Thus, CAD can weaken the heart muscles, which may lead to HF [26]. One of the methods of treating blocked or narrowed arteries is to bypass the blockage using a piece of healthy blood vessel from somewhere else in the body, which is called coronary artery bypass graft surgery [27]. In addition, aortic value stenosis is a disease in which the opening of the aortic valve narrows, restricting blood flow from the left ventricle to the aorta. It causes the heart’s left ventricle to pump harder to push blood through the narrowed aortic valve, which may lead to HF if not treated properly [28].

We also found evidence in the literature claiming that kidney failure causes HF. Dhingra’s group revealed that kidney disease places men at a higher risk of developing HF, even without diabetes or high blood pressure [29]. Hyperuricemia (although not included in the list of the overall predictive factors) is one of the links between kidney failure and HF. Reduced uric acid excretion due to kidney disease can lead to hyperuricemia, which is an elevated level of uric acid in the blood [30]. Subsequently, elevated uric acid levels are known to be one of the risk factors for HF [31,32]. Gout, identified as one of the overall predictive factors, also represents a prominent symptom associated with elevated levels of uric acid [33].

Evidence in the literature for other risk factors such as pneumonia and anemia is also available. Regarding the relationship between pneumonia and HF, Eurich et al.’s group showed that pneumonia significantly increases the risk of HF across a range of ages and severity of cases, which is the same consequence as that found in this study [34]. We also found research papers reporting the relationship between anemia and HF. It is known that anemia is one of the common comorbidities that often coexists in patients with heart failure, and is associated with poor clinical outcomes. Despite many studies on the relationship between HF and anemia, it is not entirely clear whether anemia is merely an indicator of HF severity or a mediator of HF progression [35,36]. The results of this study allow us to consider the possibility that anemia acts as one of the causes of HF.

## 5. Discussion and Conclusions

The objective of this study is to identify medical histories that could predict the progression of heart failure in patients with hypertension. However, upon analysis, it was observed that many direct predictive factors for heart failure, independent of hypertension, were prominently identified, such as coronary artery disease, kidney failure, hyperlipidemia, and atrial fibrillation. Nevertheless, factors with a less direct association with heart failure, such as pneumonia, hyperthyroidism, and anemia, were also extracted, prompting a consideration of whether these factors elevate the risk of developing heart failure due to hypertension.

The basic strategy is to identify items in a medical history that can predict the progression of disease B in the presence of disease A. In this study, for the purpose of ensuring a stable and robust analysis, disease A was defined as hypertension and disease B was selected as heart failure, given its substantial patient population. However, as hypertension and heart failure are extensively studied diseases, the analysis predominantly revealed well-established results. With the acquisition of more medical data, it would be useful to apply the analysis designed here to other diseases that are less well explored and, hence, more intriguing.

When examining the AUCs of XGBoost models, it can be observed that the overall performance was not strong. One possible reason for the low AUC may be the omission of medical conditions occurring after hypertension, which may be crucial information for the onset of heart failure. Only medical history recorded before hypertension diagnosis was utilized for the purpose of this study, which is to identify the predictive factors at the point of hypertension diagnosis.

We believe that adding dietary habit data and analyzing it comprehensively could be a beneficial research strategy. In addition, incorporating hypertension treatment data will also further strengthen this research. Specifically, utilizing the treatment data to examine cohorts receiving appropriate treatment for hypertension alongside those not receiving such treatment would enable a more targeted analysis. We anticipate that these data will be well constructed and effectively leveraged in the future.

In this investigation, we uncovered predictive factors of heart failure progression in hypertensive patients, utilizing medical diagnosis data from the MIMIC-IV database. Employing two analytical methodologies, chi-squared tests and XGBoost modeling, we generated age-specific and ICD system-specific predictive factors. Ultimately, our investigation unveiled 21 overall predictive factors. We anticipate that these findings will provide valuable insights for the risk assessment of heart failure in hypertensive patients.

## Figures and Tables

**Table 1:** The number of patients in H0 and HF classes for each subgroup.

	Subgroup	N. of HF	N. of H0	N. of Total
ICD-9	AL	1389 (4.95%)	26,700	28,089
	G1	303 (2.32%)	12,737	13,040
	G2	520 (5.72%)	8574	9094
	G3	566 (9.51%)	5389	5955
ICD-10	AL	789 (3.81%)	19,909	20,698
	G1	215 (2.43%)	8624	8839
	G2	328 (4.20%)	7478	7806
	G3	246 (6.07%)	3807	4053

## Data Availability

Python implementations are available at https://github.com/jmjung83/predictive_factor_for_HF (accessed on 22 May 2024).

## Supplementary Materials

The following supporting information can be downloaded at: https://www.mdpi.com/article/10.3390/bioengineering11060531/s1, Figure S1: Basic statistics of the preprocessing data for patients using the ICD-10 system; Figure S2: The boxplots of APRs (Average Precision Rate) of the 1000 trained XGBoost models; Table S1: ICD codes associated to heart failure; Table S2: The selected features for analysis process (ICD9); Table S3: The selected features for analysis process (ICD10); Table S4: The predictive factors by chi-square tests (ICD9); Table S5: The predictive factors by chi-square tests (ICD10); Table S6: The predictive factors by XGBoost modeling (ICD9); Table S7: The predictive factors by XGBoost modeling (ICD10); Table S8: The 21 overall predictive factors.
