# Supplementary material for: Exploring Predictive Factors for Heart Failure Progression in Hypertensive Patients Based on Medical Diagnosis Data from the MIMIC-IV Database
Source: Bioengineering (Basel). 2024 May 23;11(6):531. doi: 10.3390/bioengineering11060531 (PMC11200608; doi:10.3390/bioengineering11060531)
Supplement: Supplementary file 1 [file bioengineering-11-00531-s001.zip › supplement_Figure_S1~S2_bioeng3005587_Rev.pdf]

Exploring Predictive Factors for Heart Failure Progression in Hypertensive Patients Based on Medical Diagnosis Data from the MIMIC-IV Database

Jinmyung Jung [a,*], Doyoon Kim [a], Inkyung Hwang [a],

[a]Division of Data Science, College of Information and Communication Technology, The University of Suwon, Hwaseong 18323, Republic of Korea

* Corresponding author. E-mail address: jmjung@suwon.ac.kr

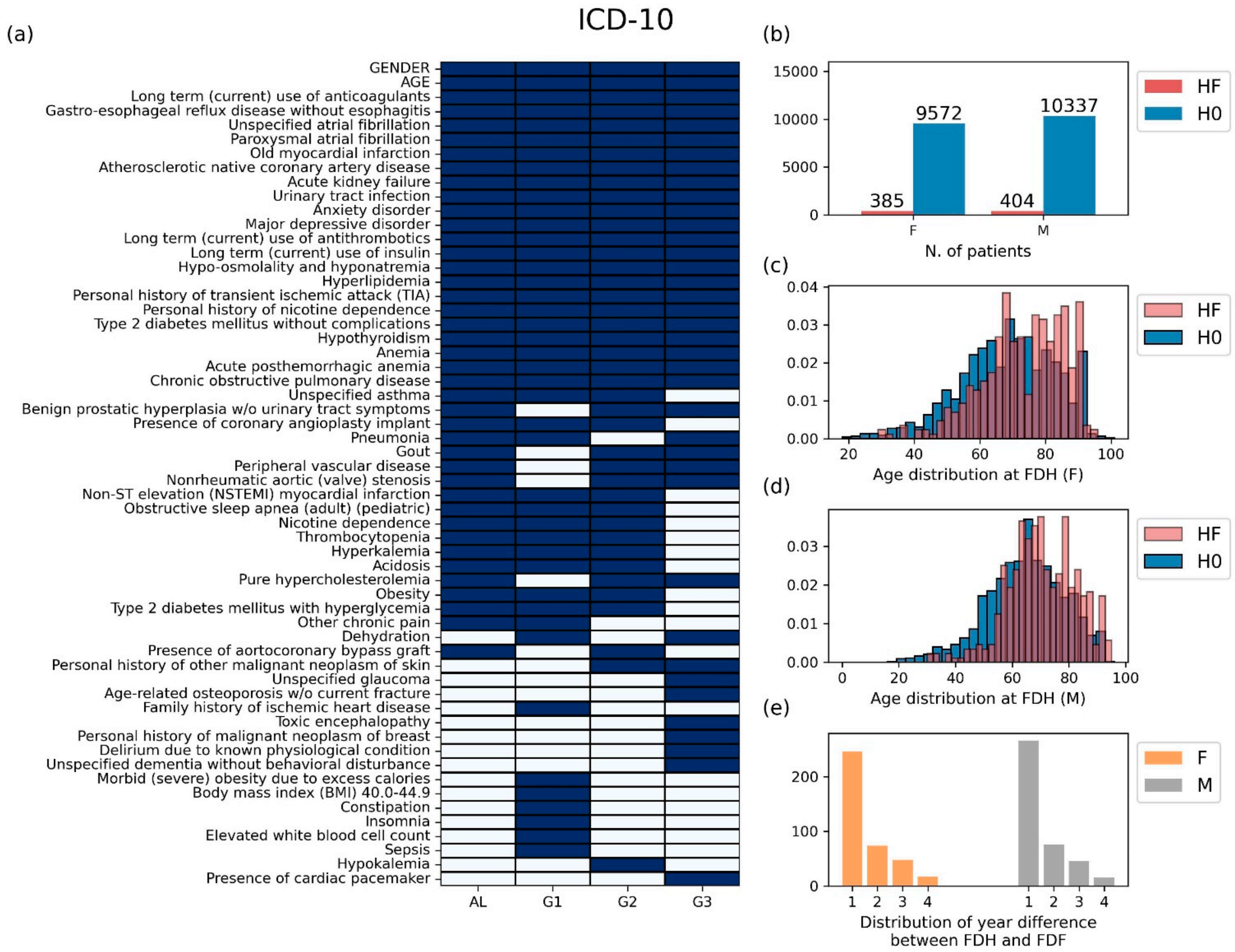

Figure S1. Basic statistics of the preprocessing data for patients using the ICD-10 system. (a) The selected features of each group for analysis process. **(b)** The number of patients by gender. **(c-d)** The distribution of ages at the first diagnosis of hypertension (FDH) for (c) male patients and (d) female patients. **(e)** The distribution of year difference between FDH and FDF. HF: group of patients diagnosed with heart failure after hypertension, H0: group of patients without heart failure after hypertension, FDH: age at the first diagnosis of hypertension, FDF: age at the first diagnosis of heart failure.

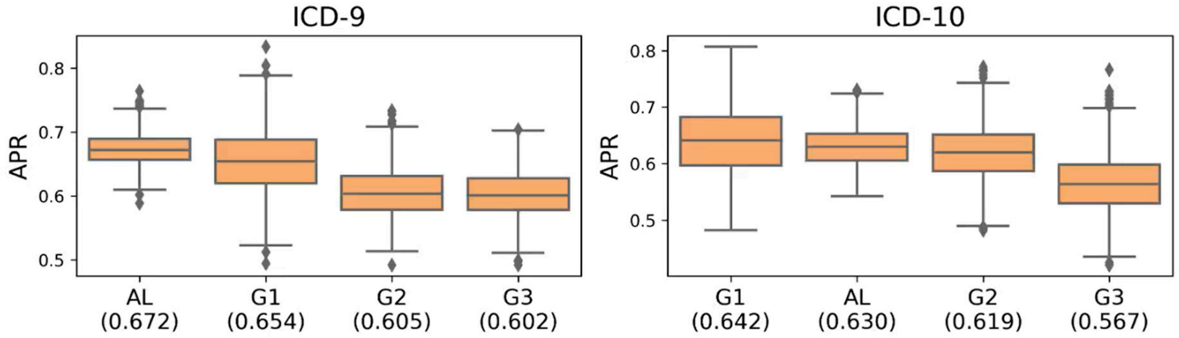

Figure S2. The boxplots of APRs (Average Precision Rate) of the 1,000 trained XGBoost models. For each subgroup, the APRs of the 1,000 trained XGBoost models are shown as a boxplot along with their averages.
